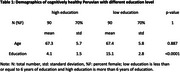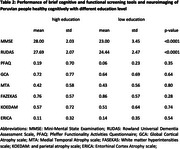# Educational Disparities and Lower Cognitive Performance on MMSE and RUDAS Despite Neuroanatomical and Functional Preservation in Aging: Insights from Peru

**DOI:** 10.1002/alz70857_104841

**Published:** 2025-12-25

**Authors:** Gregory Brown, Diego Bustamante‐Paytan, Maria Fe Albujar‐Pereira, José Carlos Huilca, Katherine Agüero, Graciet Verastegui, Zadith Yauri, Pamela Bartolo, Daniela Bendezu, Karol Melissa Lipa‐Pari, Rosa Montesinos, Agustin Ibanez, Nilton Custodio

**Affiliations:** ^1^ Instituto Peruano de Neurociencias, Lima, Lima, Peru; ^2^ University of California, San Francisco, San Francisco, CA, USA; ^3^ Equilibria, Lima, Lima, Peru; ^4^ Hospital Nacional Cayetano Heredia, Lima, Lima, Peru; ^5^ Universidad de San Martín de Porres, Facultad de Medicina, Centro de Investigación del Envejecimiento, Lima, Lima, Peru; ^6^ Unidad de Investigación y Docencia, Equilibria, Lima, Peru., Lima, Lima, Peru; ^7^ Latin American Brain Health Institute (BrainLat), Universidad Adolfo Ibañez, Santiago, Chile; ^8^ Global Brain Health Institute (GBHI), University of California San Francisco (UCSF); & Trinity College Dublin, Dublin, Ireland

## Abstract

**Background:**

Cognitive assessment of older adults who are either illiterate or with low levels of education is particularly challenging because several battery tasks require a certain educational background, despite a large portion of the world having not completed primary school. The Mini‐Mental State Examination (MMSE) and Rowland Universal Dementia Assessment Scale (RUDAS) are two widely used cognitive screening tests to identify cognitive impairments in clinical settings, but they may not be valid across diverse educational and cultural backgrounds. Furthermore, it remains unclear if the association of education with cognitive impairment is due to pathological processes or simply a failure of cognitive evaluations. We aimed to assess cognitive assessments and structural imaging in cognitively functional healthy individuals with both high and low education levels from an urban region of Peru.

**Methods:**

We recruited a total of 90 cognitively healthy individuals with low education ( £6 years, mean = 4.1) and 90 individuals with higher education (>6 years, mean = 15.1), who were matched on age and sex (table 1). We assessed individuals with CDR = 0 and the Peruvian version of MMSE. Study participants underwent a standardized volumetric T1‐ and T2‐weighted MRI protocol with rapid gradient echo and fluid attenuation inversion recovery sequences. A trained neuroradiologist assed neurodegeneration using the following scales: global cortical atrophy (GCA), entorhinal cortex atrophy (ERICA), medial temporal atrophy (MTA), and parietal atrophy (KOEDAM), and the FASEKAS scale was used to quantify white matter hyperintensities.

**Results:**

Low education individuals scored much lower on MMSE, compared to high education individuals (*p* <0.001), despite having similar metrics on MRI (*p* >0.28) and similar levels of impairments on activities of daily living (*p* = 0.35, table 2).

**Conclusion:**

Our findings show that urban Peruvians with low education score significantly lower on the MMSE and RUDAS, even with similar age, sex, brain structure, and function. This highlights the limitations of these assessments in accounting for educational disparities, potentially misclassifying healthy individuals as impaired. These results emphasize the need for culturally and educationally adapted cognitive screening tools to ensure accurate assessments in diverse populations. More work is needed to identify brief, culturally appropriate cognitive assessments validated for varying educational levels.